# The Pulsation of Veins

**Published:** 1883-06

**Authors:** 


### ARTICLE III.

### THE PULSATION OF VEINS.

The classic venous pulse consists of a regurgitant wave. It is due to insufficiency of the valves of the veins and of the auriculo-ventricular valves of the right side of the heart. With this insufficiency, the ventricular systole causes not only a forward impulse to the blood in the pulmonary artery, but also a backward impulse to the blood in the auricle, vena cava, jugular, and even in more peripheral veins.

Formerly no other venous pulse was recognized, the presence of any pulsation in veins being considered pathognomic of regurgitation through the bicuspid valves. Two other varieties, however, exist. One of these varieties is due to a relaxation of the minute arteries, which relaxation allows a projection of the arterial waves through the capillaries into the veins.

In Bernard's experiment upon the submaxillary gland by irritation of the chorda tympani, the arterioles become dilated, pulsations may be seen in the capillaries, and the blood issues in jets from the vein.

Quircke has reported several cases of pulsation in the veins of the hand which ceased upon the compression of the artery, but upon the compression of the vein ceased only on the proximal side. Here, also, the arterial wave must have been carried through the capillaries, as in the experiment with the submaxillary gland.

Pulsatory movement of the venous blood may occur, therefore, from a forward impulse through the capillaries, and also from a backward impulse, which proceeds directly from the heart. The first is most marked at the periphery, and the second is most marked at the center of the venous system. Both, however, are positively systolic in both ; the wall of the vein rises at the same time as does the arterial wall. The third variety of venous pulsation is distinguished from those we have considered by the fact that it is negatively systolic. This venous pulse contracts with that of the artery in a prolonged ascent, and a descent which is abrupt; also in the fact that the descent of the one and the ascent of the other are simultaneous. The vein therefore falls while the artery rises, and rises while the artery falls.

The abrupt descent is in this venous pulse the feature which is most marked. Moss, in 1876 (Die Diagnostik des Pulses), named it the negative pulse of the veins.

Modern interest in the subject commences with this date.

In 1828, however, Hedmeyer reported pulsatory oscillations in the fluid of a tube, one end of which was emersed in water, the other end being inserted into the internal jugular of the horse.

Meyrich, in 1845, repeated the experiment upon dogs. He found the fluid to rise during the systole, and to fall during the diastole of the heart. According to Kallett (Hermann's Hdb. du Phys. Bd. iv. Th. 1), Meyrich ascribed the periodic ascent to the aspiratory influence of the auricle at the moment of its relaxation. Mosso supposed it to be due to a periodic increase in the aspiration of the thorax. The plethysmograph demonstrates that the heart occupies less space during its systole than during its diastole, also with a manometer in the mouth it has been noted that the mercury with suspension of the respiration falls during the systole and rises during the diastole of the heart. It was upon these data that Mosso's conclusion was based.

Gottwall, however, in 1881 (*Arch. f. d. Ges. Phys.*), with experiments upon dogs, found the pulse to persist after the openng of the thorax ; he, therefore, considered it directly dependent upon the heart. Riegel (*Berl. Klin Wochenschrift*, March 2, 1881), revised the theory of Meyrich. Relaxation of the auricle is syncronous with the contraction of the ventricle ; the fall of venous pressure is, therefore, said to be syncronous with the systole of the heart. Riegel, in May of 1882 (*Deutches Arch. für Klin Med.*), issued a series of tracings taken simultaneously from the carotid artery, and from the jugular vein. These tracings corroborate that coincidence in time which has been referred to.

The ascent of the vein was proved to be diastolic, and the descent to occupy the time of the arterial percussion stroke. Francois Franck, in April of 1882 (*Gaz. Hebd. de Med.*), gave simultaneous tracings from the heart and from the vein. These tracings show further that the descent of the first smaller wave accompanies the relaxation of the ventricles. Following the cardiac cycle of events, he finds the ascent of the apex due to the accumulation of blood from obstruction to its discharge during the contraction of the auricle; the sudden descent to the aspiratory influence of the relaxation of the auricle; the small descent to retardation from the loss of that influence; the descent to the aspiration of the ventricle, and the prolonged ascent to the gradual repletion of the whole right heart.

The literature of this subject has been comprised of the writings of Mosso, of Gottwalt, of Regil and of Franck.

By none of these experimentors has the pulse been obtained beyond the jugulars, excepting once by Gottwalb in a bronchial vein. Recently it has been obtained by Dr. Sarah E. Post, from Milwaukee. Also in veins of the forearm and in the foot (N. Y. Med. Record, Feb. 17, 1883). Dr. Post has not only traced this pulse farther per-pharally than has any previous observer, but she is also second only to Marcey in the registration of any kind of a venous pulse in the lower limb. It is interesting to note that the instrument used by Dr. Post is American in its origin and make. The lady concludes as follows: "The recognition of this pulse is important because it may be of sufficient amplitude to mark the trace of the arterial pulse, because it must be eliminated in the diagnosis of the pulse of regurgitation through the tricusgid valve and because it probably possesses a diagnostic importance of its own. Its recognition with the Pond syhygmograph is not difficult. Having obtained the radial arterial pulse, move the instrument about $\frac{1}{2}$ c. m. toward the exterior side of the arm, remit one-half the pressure, and if it be present its tracing will appear. Complaint regarding the unreliability of the Pond sphygmograph have originated in the fact that it so readily registers this pulse. Not being recognized it has been considered a vagary of the little instrument itself."

Abroad, this pulse is commonly called the normal venous pulse.

In the radial vein, Dr. Post considers it to have a pathological significance. As she has found it only in connection with venous engorgement from arterial relaxation, or from insufficiency in the heart, she supposes it to be extended and accented by that condition, when associated with sufficient cardiac power. It always disappears with the failure of the heart.

---

SKEPTICISM IN MEDICINE.—Certainly he who has not faith, and does nol use in the spirit of faith, properly guided by proper consideration and an ever increasing experience, opium, ipecacuanha, mercury, arsenic, antimony, ergot, iron, zinc, phosphorus, bismuth and their preparations, chloral, croton chloral, aconite, belladonna, sp. eth. nitrosi, sp. eth. sulphurici, quinine, iodide, and bromide of potassium, and many other drugs, can never be a thoroughly successful practitioner in the curative results of his practice, nor can we believe him to be a happy one, if possessed of a conscience, since he must see many cases, that in other hands may do well, go from bad to worse, and end fatally in his. Instead of lowering the estimation in which our materia medica is held, increased knowledge is likely to carry it to a still more honored position, when the powers of each drug, from accurate observation, become more thoroughly defined.—*The Medical Press.*

A MICRO.ORGANISM FOR YELLOW FEVER.—Peronospera Lutea is the title of the Micro-Organism which Dr. Carmona Del Valle believes to be characteristic of yellow fever. The germ of this cryptogerm are always to be found in the excretions and in the fluids and secretions. Animals inoculated with it show febrile symptoms. After recovery they are not affected by a second inoculation.—*The Medical Record.*

DEATH OF A LEPROUS PATIENT AT SALEM, MASS.—Charles D. Erby, a leprous patient at the Salem (Mass.) Almshouse, who contracted the disease in the Sandwich Islands, and whose case has excited much apprehension, died there March 19th.—*The Medical Record.*

